# Study on the High-Temperature Microwave Absorption Performance and Mechanism of SiC Nanowire-Reinforced Porous Si_3_N_4_ Ceramics

**DOI:** 10.3390/ma18174071

**Published:** 2025-08-30

**Authors:** Jialin Bai, Xiumin Yao, Xuejian Liu, Zhengren Huang

**Affiliations:** 1 State Key Laboratory of High Performance Ceramics and Superfine Microstructures, Shanghai Institute of Ceramics, Chinese Academy of Sciences, Shanghai 200050, China; baijialin21@mails.ucas.ac.cn; 2 Center of Materials Science and Optoelectronic Engineering, University of Chinese Academy of Sciences, Beijing 100049, China

**Keywords:** SiC_nw_/Si_3_N_4_ ceramics, high temperature, electromagnetic wave absorption, dielectric properties

## Abstract

SiC nanowires (SiC_nw_), due to their excellent dielectric properties, are promising high-temperature absorbing materials. However, the mechanism of their high-temperature absorption still requires further research. Therefore, porous SiC_nw_/Si_3_N_4_ and SiC/Si_3_N_4_ ceramics with different SiC phase morphologies were fabricated using a simple precursor impregnation and pyrolysis method. The Fe impurity content of the Si_3_N_4_ powder raw material significantly affects the generation of SiC nanowires. When SiC exists in the form of nanowires, the excellent conductivity brought by the conductive network of the nanowires causes a significant response of the material’s permittivity to temperature. When the test temperature is room temperature, SiC_nw_/Si_3_N_4_ has excellent absorption performance with a minimum reflection loss of −29.75 dB at 2.16 mm and an effective absorption bandwidth of 3.72 GHz at 2.54 mm. As the test temperature increases to 300 °C, the effective absorption bandwidth of SiC_nw_/Si_3_N_4_ covers the entire X-band. The porous SiC_nw_/Si_3_N_4_ ceramics exhibit excellent electromagnetic wave absorption performance, demonstrating significant application potential for high-temperature environments.

## 1. Introduction

When a stealth aircraft flies at high speeds, friction with the air generates heat, causing a sharp rise in temperature on certain surfaces of the aircraft. This may result in the loss of stealth properties of the materials [1,2]. An ideal electromagnetic wave (EMW) absorbing material should not only adhere to the design principles of thin, light, wide, and strong, but also exhibit strong resistance to high temperatures and oxidation [3,4,5].

Carbon materials (e.g., carbon fiber [6], graphene [7], carbon nanotubes [8], and carbon black [9]) and magnetic materials (e.g., Fe_3_O_4_ [10], Co [11], and Ni [12]) cannot be used in complex and harsh environments, such as high temperature and oxygen content, due to poor oxidation resistance (typically below 500 °C [13]) or lost magnetism above the Curie temperature and other factors. Ceramic-based materials show great potential for high-temperature microwave absorption because of their excellent high-temperature resistance [14], corrosion resistance [15], low density [16], oxidation resistance [17,18], and adjustable dielectric properties [19]. Excellent microwave absorption performance needs to take into account the impedance matching performance and attenuation performance [20]. Dispersing the wave-absorbing phase in the wave-transmitting phase to form A/B [21] or A/B/C [22,23] type absorbing material is an effective method to make the EMW enter the material as much as possible and achieve efficient absorption. Si_3_N_4_ ceramics are an ideal wave-transmitting substrate material due to their high-temperature resistance and low dielectric constant [24].

SiC is widely used in the high-temperature EMW absorption due to its excellent high-temperature resistance and adjustable dielectric properties [25]. However, the high resistivity and single loss mechanism of SiC result in poor EMW absorption performance [19]. In contrast to the isoaxial SiC particles, SiC nanowires (SiC_nw_) exhibit superior electrical properties and a unique one-dimensional structure, thus demonstrating more outstanding performance in EMW absorption [26,27]. Han et al. [28] achieved a hierarchical network composed of graphene/SiC nanowires within a SiOC matrix through the polymer pyrolysis process. At 400 °C, the effective absorption bandwidth (EAB) of this network reached 3.9 GHz. Although SiC nanowires have made some progress for high-temperature EMW absorption, the absorption mechanisms in multi-component systems remain complex. A significant challenge is quantifying the individual contribution of each material to the overall high-temperature absorption performance. Therefore, the intrinsic high-temperature absorbing mechanism of SiC nanowires still requires further investigation.

In this study, SiC_nw_/Si_3_N_4_ and SiC_nw_/Si_3_N_4_ ceramics with different SiC phase morphologies were prepared using two different purities of Si_3_N_4_ powder as raw materials through a combination of gas pressure sintering and precursor impregnation pyrolysis. The microstructure and dielectric properties of SiC with different morphologies were systematically investigated. Through comparative analysis of the permittivity of SiC phases with varying morphologies, the high-temperature microwave absorption mechanism of SiC nanowires was elucidated. This work provides certain references for the application research of SiC nanowires in high-temperature EMW absorption.

## 2. Experimental Procedure

### 2.1. Materials

Two types of Si_3_N_4_ powder (d_50_ = ~1 μm, A-type: purity 98%, B-type: purity 99.5%) with different iron impurity contents were used as starting materials (Table 1). Y_2_O_3_ (d_50_ = ~1 μm, purity 99.9%, Aladdin, Shanghai, China) and Al_2_O_3_ (d_50_ = ~1 μm, purity 99.9%, Aladdin, Shanghai, China) were used as sintering additives. Mono-dispersed poly methyl methacrylate (PMMA, d_50_ = ~30 μm, purity 99.9%, Dongguan Kemai New Materials Co., Ltd., Dongguan, China) micro-balls were used as a pore-forming agent. Vinyl hydrogen-containing polycarbosilane (PCS, purity 99%, Institute of Chemistry, Chinese Academy of Sciences, Beijing, China) was used as the preceramic. All the chemicals were used as received without further purification.

### 2.2. Preparation of Porous SiC_nw_/Si_3_N_4_ and SiC/Si_3_N_4_ Ceramics

The sintering process of porous Si_3_N_4_ ceramics has been reported in detail in previous work [29,30]. The porous Si_3_N_4_ ceramic sintered from low-purity A-type Si_3_N_4_ powder was placed in PCS solution with a concentration of 25 wt% for 30 min and then transferred together to a vacuum chamber for 30 min to allow the solution to fill the internal pores of the ceramic. Finally, the Si_3_N_4_ ceramic impregnated with PCS solution was placed in a tube furnace and successively cross-linked at 200 °C for 2 h, pyrolyzed at 900 °C for 2 h, and heat-treated at 1500 °C for 2 h to obtain SiC_nw_/Si_3_N_4_ ceramics. The atmosphere in the tubular furnace is argon. Similarly, porous SiC/Si_3_N_4_ ceramics were obtained by impregnating, crosslinking, pyrolyzing, and heat treating porous Si_3_N_4_ ceramics sintered from B-type Si_3_N_4_ powder.

### 2.3. Characterization

X-ray diffraction (XRD, D8 ADVANCE, Bruker Corporation, Karlsruhe, Germany) was conducted to determine the phase composition. A field emission scanning electron microscope (SEM, SU8220, Hitachi High-Tech Corporation, Tokyo, Japan) was employed to characterize the microstructure. Inductively coupled plasma mass spectrometry (ICP-MS, JY2000-2, HORIBA Group, Kyoto, Japan) was utilized to evaluate the Fe content. The relative complex permittivity in the X-band was measured using a vector network analyzer (VNA, Agilent E5071C, Keysight Technologies, Santa Rosa, CA, USA). The sample dimensions were 22.86 mm × 10.16 mm × 3 mm.

The EMW absorption performance is determined by calculating the reflection loss (*RL*), as shown in the following equations [31]:

(1) $$RL=20\log_{10}\frac{\left|Z_{in}-Z_{0}\right|}{\left|Z_{in}+Z_{0}\right|}$$

(2) $$Z_{in}=Z_{0}\sqrt{\frac{\mu_{r}}{\varepsilon_{r}}}tanh[j\frac{2\pi fd}{c}\sqrt{\mu_{r}\varepsilon_{r}}]$$

where $Z_{in}$ is the input impedance and $Z_{0}$ is the impedance of the EMW in free space (377 Ω). $\mu_{r}$ ($\mu_{r}=\mu^{\prime }-{j\mu }^{\prime \prime }$) is the relative complex permeability of the samples. $\varepsilon_{r}$ ($\varepsilon_{r}=\varepsilon^{\prime }-{j\varepsilon }^{\prime \prime }$) is the relative complex permittivity. *f*, *c*, and *d* are the frequency, light speed, and thickness, respectively. The material involved in this paper is non-magnetic, so $\mu^{\prime }$ and $\mu^{\prime \prime }$ are taken as 1 and 0, respectively.

## 3. Results and Discussion

### 3.1. Synthesis and Characterization

**Scheme 1:**
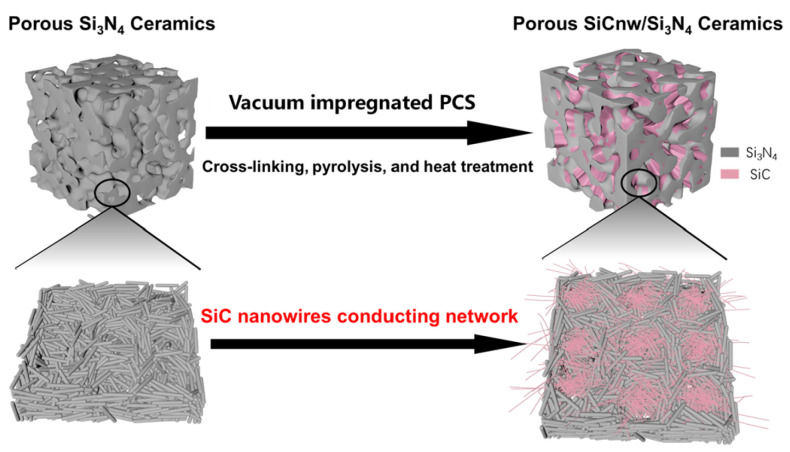
Schematic diagram of the preparation of porous SiC_nw_/Si_3_N_4_ ceramics.

As shown in Scheme 1, SiC_nw_/Si_3_N_4_ composite ceramics were prepared by vacuum impregnating PCS on porous Si_3_N_4_ ceramics, followed by cross-linking, pyrolysis, and heat treatment. The permittivity of porous Si_3_N_4_ ceramics is very low, which promotes more EMW into the material to interact with the SiC nanowires [16]. As the precursor of SiC ceramics, PCS will gradually precipitate SiC nanocrystals from the amorphous phase during pyrolysis and heat treatment [32].

**Figure 1:**
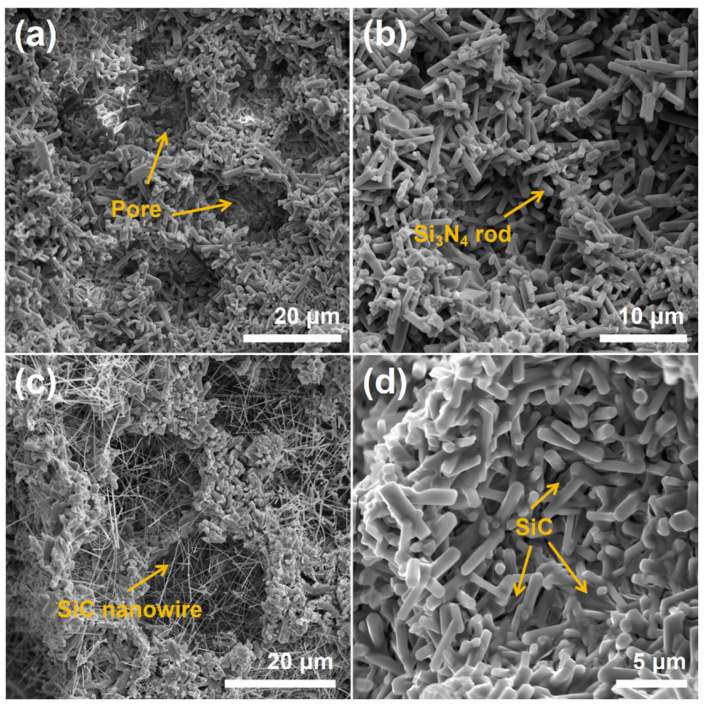
SEM image of ceramic fracture surface: (**a**,**b**) Porous Si_3_N_4_ and (**c**) SiC_nw_/Si_3_N_4_ ceramics prepared using A-type Si_3_N_4_ powder. (**d**) Porous SiC/Si_3_N_4_ ceramics prepared using B-type Si_3_N_4_ powder.

The morphology of the samples is shown in Figure 1. The results show that the pore diameter of the porous Si_3_N_4_ ceramic is approximately 17 μm, and it is composed of rod-like structures, with a porosity of 64.3%. Figure 1c shows that in the Si_3_N_4_ ceramic pores with a higher content of iron impurities, PCS transforms into SiC nanowires after pyrolysis and heat treatment. Under the action of the metal catalyst, SiO and CO gases generated by the pyrolysis of PCS are dissolved in metal droplets to form SiC nanocrystals and grow in the direction that is conducive to thermodynamics, and then gradually form SiC nanowires [33]. In contrast, PCS did not transform into SiC nanowires in Si_3_N_4_ ceramics prepared from higher-purity B-type Si_3_N_4_ powder but instead distributed as SiC layers on the pore walls. SiC phases with different morphologies and structures were grown in Si_3_N_4_ ceramics obtained from different types of Si_3_N_4_ powders under the same processing conditions. Subsequently, by comparing the dielectric properties of SiC_nw_/Si_3_N_4_ and SiC/Si_3_N_4_, it will be more conducive to revealing the high-temperature wave absorption mechanism of SiC nanowires.

**Figure 2:**
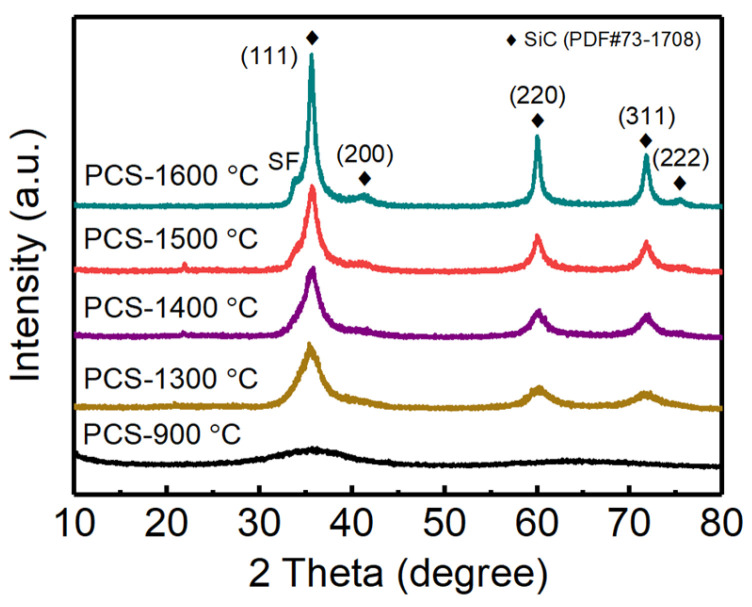
XRD patterns of PCS at different heat treatment temperatures.

Figure 2 presents the XRD patterns of PCS at different heat treatment temperatures from 900 °C to 1600 °C in an Ar atmosphere for 2 h. The sample pyrolyzed at 900 °C shows weak peak intensity and broad peak width, suggesting low crystallinity. After heat treatment at 1300~1600 °C, distinct diffraction peaks appeared at 35.7°, 41.5°, 60.1°, 72.0° and 75.7° corresponding to the (111), (200), (220), (311), and (222) planes of 3C-SiC (F-43m) with a cubic crystal structure (PDF#73-1708), respectively. The intensity of the diffraction peaks increases with the rise of the heat treatment temperature, indicating that the crystallinity of SiC has improved. Based on the Scheeler formula, the average crystal size of SiC at 1300 °C, 1400 °C, 1500 °C, and 1600 °C was calculated to be 5.0 nm, 6.8 nm, 7.7 nm, and 13.3 nm, respectively, meaning that the average crystal size increases correspondingly with the increase in temperature. Therefore, the SiC phase in SiC/Si_3_N_4_ ceramics is mainly composed of amorphous and nanocrystalline states.

### 3.2. Dielectric and High-Temperature EMW Absorption Performance

**Figure 3:**
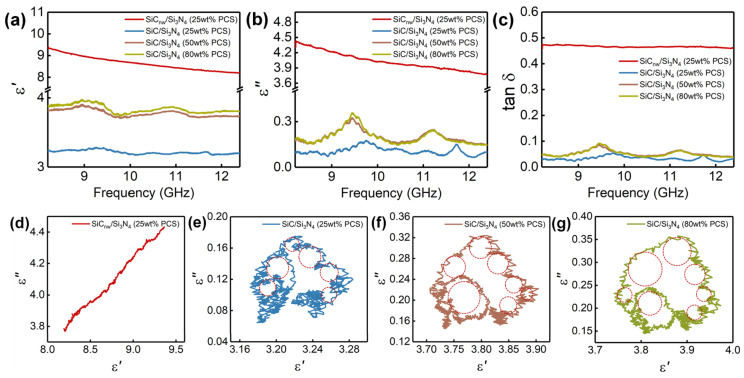
(**a**) The $\varepsilon^{\prime }$, (**b**) $\varepsilon^{\prime \prime }$, (**c**) dielectric loss tangent (tanδ), and (**d**–**g**) Cole–Cole plots of SiC_nw_/Si_3_N_4_ and SiC/Si_3_N_4_.

The complex permittivity of SiC_nw_/Si_3_N_4_ and SiC/Si_3_N_4_ composite ceramics was tested at X-band (Figure 3a–c). The real part of complex permittivity ($\varepsilon^{\prime }$) stands for the capacity to store electromagnetic energy, while the imaginary part ($\varepsilon^{\prime \prime }$) indicates the inner dissipation [34,35]. For SiC_nw_/Si_3_N_4_ composite ceramics, both $\varepsilon^{\prime }$ and $\varepsilon^{\prime \prime }$ show obvious frequency dispersion [36]. As the frequency increases, the values of $\varepsilon^{\prime }$ and $\varepsilon^{\prime \prime }$ gradually decrease, which is beneficial for broadband absorption. The $\varepsilon^{\prime }$ value of SiC_nw_/Si_3_N_4_ ranges from 8.20 to 9.35, and the $\varepsilon^{\prime \prime }$ value ranges from 3.77 to 4.42. Compared with SiC_nw_/Si_3_N_4_, the $\varepsilon^{\prime }$ value of SiC/Si_3_N_4_ obtained under the same 25 wt% concentration of PCS in the X-band is all less than 4, and the $\varepsilon^{\prime \prime }$ value is less than 0.3. The complex permittivity of SiC/Si_3_N_4_ is significantly lower than that of SiC_nw_/Si_3_N_4_, and its dielectric loss intensity is low (Figure 3c), making it unable to effectively absorb EMW. Although the concentration of PCS can be increased to raise the SiC content in SiC/Si_3_N_4_, the dielectric loss intensity cannot be effectively improved. This is because the crystallinity of SiC in SiC/Si_3_N_4_ is low, resulting in low electrical conductivity. It is worth noting that the permittivity curve of SiC/Si_3_N_4_ has multiple distinct relaxation peaks. This is because the SiC layer is uniformly distributed on the pore walls of the porous Si_3_N_4_ ceramic, forming a large number of SiC-Si_3_N_4_ interfaces, which leads to the occurrence of polarization relaxation phenomena [29].

The influence of polarization relaxation loss dominated by defects and interfaces and conduction loss dominated by conductivity on the dielectric loss intensity can be revealed through the Cole–Cole plots [37]. Each semicircle corresponds to a Debye relaxation process, indicating that the EMW dissipation is attributed to the relaxation polarization loss [38]. The straight line part of the Cole–Cole curve implies the role of the conduction loss [39]. Figure 3d shows that the semi-circular shape of SiC_nw_/Si_3_N_4_ is very small, and the overall trend is a straight line, indicating that the intensity of conduction loss is significantly higher than that of polarization loss. For SiC/Si_3_N_4_, the Cole–Cole curve consists of multiple semi-circles but no linear part (Figure 3e–g). This suggests that EMW absorption is dominated by the interface between the SiC layer and the Si_3_N_4_, rather than by the material’s conductivity, which is comparatively poor. Under the influence of the electromagnetic field, charges tend to accumulate at the SiC-Si_3_N_4_ heterojunction interface, thereby causing interface polarization loss [40]. A comparison of the Cole–Cole curves and dielectric loss intensities of SiC_nw_/Si_3_N_4_ and SiC/Si_3_N_4_ reveals the superior electrical conductivity of SiC nanowires over SiC nanocrystalline. In the dielectric loss mechanism of SiC_nw_/Si_3_N_4_, the electrical conductivity plays a decisive role.

**Figure 4:**
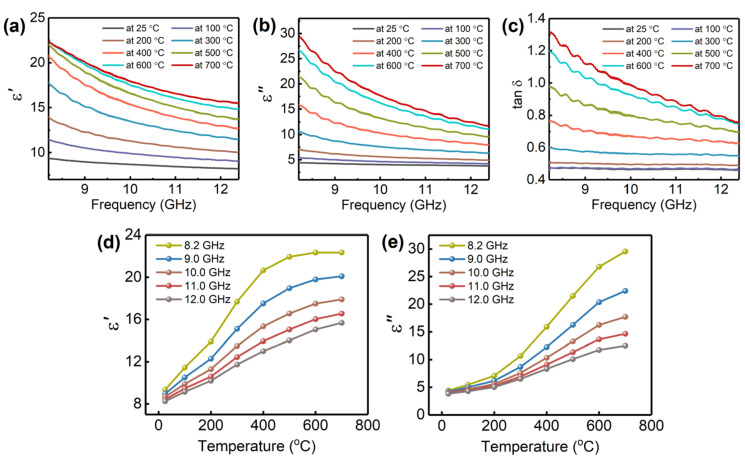
The frequency-dependent curves of (**a**) $\varepsilon^{\prime }$, (**b**) $\varepsilon^{\prime \prime }$, and (**c**) tanδ for SiC_nw_/Si_3_N_4_ in the temperature range of 25–700 °C. The (**d**) $\varepsilon^{\prime }$ and (**e**) $\varepsilon^{\prime \prime }$ curves of SiC_nw_/Si_3_N_4_ as a function of temperature at different frequencies.

The complex permittivity of SiC_nw_/Si_3_N_4_ was measured from 25 to 700 °C. Figure 4a–c show that $\varepsilon^{\prime }$, $\varepsilon^{\prime \prime }$, and tanδ all exhibit significant temperature dependence. Based on the Debye relaxation theory, $\varepsilon^{\prime }$ and $\varepsilon^{\prime \prime }$ are given as follows [25,35]:

(3) $$\varepsilon^{\prime }=\varepsilon_{\infty }+\frac{\varepsilon_{s}-\varepsilon_{\infty }}{1+{\left(2\pi f\right)}^{2}{\tau (T)}^{2}}$$

(4) $$\varepsilon^{\prime \prime }=\frac{2\pi f{(\varepsilon }_{s}-\varepsilon_{\infty })}{1+{\left(2\pi f\right)}^{2}{\tau (T)}^{2}}\tau (T)+\frac{\sigma (T)}{2\pi f\varepsilon_{0}}$$

where $\varepsilon_{s}$ and $\varepsilon_{\infty }$ are static permittivity and relative permittivity at high-frequency limit. $\varepsilon_{0}$ is the relatively complex permittivity in vacuum. τ(T) and σ(T) represent the relaxation time and conductivity, respectively, which vary with temperature changes. The influence of temperature on τ(T) and σ(T) can be further expressed as follows:

(5) $$\tau \left(T\right)=\frac{1}{2\nu }e^{\frac{U}{kT}}$$

(6) $$\sigma \left(T\right)=Ae^{-\frac{B}{T}}$$

Known from Equations (3)–(6), the $\varepsilon^{\prime }$ value of the material is related to the relaxation time, while the $\varepsilon^{\prime \prime }$ value is influenced by both the relaxation time and the electrical conductivity of the material. Moreover, both the relaxation time and conductivity are strong functions of temperature. The elevation of $\varepsilon^{\prime }$ at elevated temperature can be explained by the rapid response of the electron motion to the alternating electromagnetic field, which shortens the relaxation time [41]. The rise in $\varepsilon^{\prime \prime }$ at elevated temperatures is explained by the transition of electrons from the valence band to the conduction band, which increases the conductivity [42]. As shown in e, the $\varepsilon^{\prime \prime }$ value rises more rapidly than $\varepsilon^{\prime }$ with increasing temperature, leading to enhanced dielectric loss.

**Figure 5:**
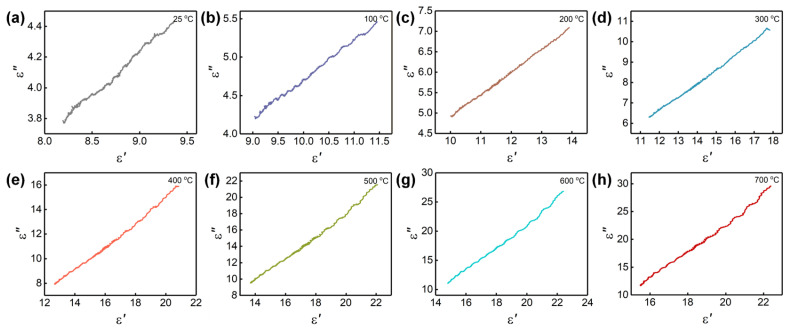
The Cole–Cole plots of SiC_nw_/Si_3_N_4_ at different temperatures: (**a**) 25 °C, (**b**) 100 °C, (**c**) 200 °C, (**d**) 300 °C, (**e**) 400 °C, (**f**) 500 °C, (**g**) 600 °C, and (**h**) 700 °C.

The influence of test temperature on the conduction loss and polarization loss of the material was further investigated, as shown in Figure 5. The results show that the Cole–Cole curves of the samples at different test temperatures all exhibited distinct straight lines, indicating that the conduction loss caused by conductivity has been playing a dominant role. Although some researchers believe that an increase in temperature can help reduce polarization loss, thereby partially balancing the increase in conduction loss and weakening the sensitivity of the material’s permittivity to temperature [43]. However, the high conductivity brought about by the conductive network formed by SiC nanowires in SiC_nw_/Si_3_N_4_ results in very little contribution of polarization loss to the absorption performance at room temperature (RT, 25 °C), thus preventing it from offsetting the increase in conduction loss under high-temperature conditions. Therefore, the permittivity of SiC_nw_/Si_3_N_4_ still increases significantly with an increase in temperature.

**Figure 6:**
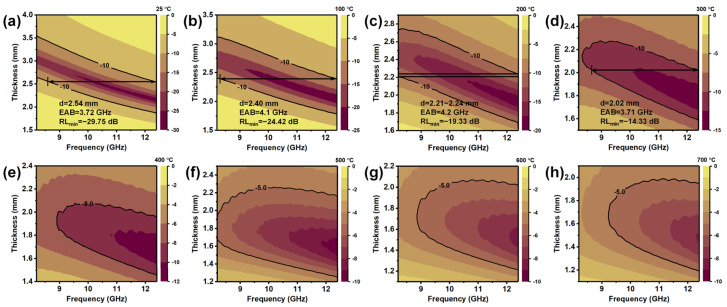
*RL* values dependence of frequency and thickness for SiC_nw_/Si_3_N_4_ at different temperatures: (**a**) 25 °C, (**b**) 100 °C, (**c**) 200 °C, (**d**) 300 °C, (**e**) 400 °C, (**f**) 500 °C, (**g**) 600 °C, and (**h**) 700 °C.

The EMW absorption performance can be more intuitively assessed by reflection loss, which is determined with the complex permittivity. Figure 6 and Table 2 show the variation of *RL* with frequency and thickness for SiC_nw_/Si_3_N_4_ measured between 25 °C and 700 °C. At 25 °C, the EAB of SiC_nw_/Si_3_N_4_ is 3.72 GHz at a thickness of 2.54 mm and the minimum reflection loss (*RL_min_*) reaches −29.75 dB (Figure 6a). When the temperature increases to 100 °C, the *RL_min_* reaches −24.42 dB, and the EAB broadens to 4.10 GHz at a thickness of 2.40 mm, accounting for 97.62% of the entire X-band (Figure 6b). Furthermore, at 200 °C, the absorption bandwidth of the material in the thickness range of 2.21 to 2.24 mm can cover the X-band (Figure 6c). The improvement in EAB is due to the increased dielectric loss and strengthened frequency dispersion effect of the complex permittivity [44]. However, high permittivity often causes impedance mismatch between the material and free space, causing more EMW to be reflected rather than absorbed at the surface of the material. When the permittivity continues to rise beyond the optimal range, the absorption performance will deteriorate. For instance, as the temperature reaches 300 °C, the absorption bandwidth narrows to 3.71 GHz (Figure 6d). At temperatures above 300 °C, the reflection loss across the entire frequency band exceeds −10 dB, resulting in an absorption bandwidth of 0 GHz (Figure 6e–h). Despite this decline at higher temperatures, the SiC_nw_/Si_3_N_4_ composite ceramics still demonstrate competitive high-temperature EMW absorption performance compared with other materials reported in previous studies (Table 3).

The high-temperature EMW absorption performance of the SiC_nw_/Si_3_N_4_ composite ceramics is attributed to multiple scattering within the pore structure and conduction loss. The low permittivity of the porous Si_3_N_4_ ceramic matrix is conducive to improving the impedance matching and promoting the EMW to enter the material, resulting in energy dissipation [51,52]. The staggered distribution of SiC nanowires within the pore structure forms a three-dimensional conductive network, which provides a prolonged transmission channel for the movement of free electrons [27]. As the environmental temperature rises, the conductivity of SiC nanowires increases, leading to enhanced conduction losses and increased permittivity. Consequently, the permittivity of SiC_nw_/Si_3_N_4_ exhibits a strong temperature response to environmental temperature, making it difficult for the material to achieve a wide absorption bandwidth across a broad temperature range.

## 4. Conclusions

In summary, porous SiC_nw_/Si_3_N_4_ and SiC/Si_3_N_4_ ceramics with different SiC morphologies were fabricated using two types of Si_3_N_4_ powders with different iron impurity contents via gas pressure sintering followed by precursor infiltration and pyrolysis. For SiC_nw_/Si_3_N_4_ ceramics, the excellent conductivity of the SiC nanowire conductive network causes conduction loss to dominate in EMW absorption. As the test temperature rises, the conduction loss caused by the electrical conductivity continuously increases, resulting in poor stability of the material’s permittivity and EMW absorption performance. The results show that SiC_nw_/Si_3_N_4_ achieved excellent absorption performance with an *RL_min_* of −29.75 dB at 2.16 mm and an EAB of 3.72 GHz at 2.54 mm when the test temperature is room temperature. By elevating the temperature, the EAB of SiC_nw_/Si_3_N_4_ covers the entire X-band at a thickness of 2.21~2.24 mm. As the permittivity continues to increase and exceeds the optimal range, the absorption performance of the material begins to decline.

## Figures and Tables

**Table 1:** The content of Fe in Si_3_N_4_ powder.

Sample	Fe (wt.%)
A-type Si_3_N_4_ powder	0.26
B-type Si_3_N_4_ powder	0.002

**Table 2:** EMW absorption properties of SiC_nw_/Si_3_N_4_ in the temperature range of 25–700 °C.

Operating Temperature (°C)	*RL*< −10 dB		*RL_min_* (dB)
	EAB (GHz)	Thickness (mm)	
25	3.72	2.54	−29.75
100	4.10	2.40	−24.42
200	4.20	2.21–2.24	−19.33
300	3.71	2.02	−17.25
400	1.46	1.81	−11.29
500	—	—	−9.51
600	—	—	−8.42
700	—	—	−8.12

**Table 3:** Comparison of the EMW absorption properties of this work with reported materials.

Material	Operating Temperature (°C)	*RL* < −10 dB		*RL_min_* (−dB)	
		EAB (GHz)	Thickness (mm)		
SiC_f_/Si_3_N_4_	RT	1.93	—	−22.55	[45]
	400	2.29	—	−24.72	
C_f_/SiCNFs/Si_3_N_4_	RT	2.3	2.6	−15.1	[46]
SiC_nw_/SiOC	RT	3.57	2.5	−17.25	[47]
C_f_/Si_3_N_4_	RT	2.23	2.0	−19.6	[48]
ZnO@MWCNTs/SiO_2_	RT	<2.7	2.5	−20.7	[49]
	400	3.4	2.5	−13.0	
SiC_f_/SiC-SiC_nw_	RT	1.3	4.5	−16.5	[50]
	600	2.8	2.5	−47.55	
SiC_nw_/Si_3_N_4_	RT	3.72	2.54	−29.75	This work
	300	3.71	2.02	−17.25	

## Data Availability

The original contributions presented in this study are included in the article. Further inquiries can be directed to the corresponding authors.
